# Optically-Powered Wireless Sensor Nodes towards Industrial Internet of Things

**DOI:** 10.3390/s22010057

**Published:** 2021-12-23

**Authors:** Letícia C. Souza, Egidio R. Neto, Eduardo S. Lima, Arismar Cerqueira Sodré Junior

**Affiliations:** Laboratory WOCA, National Institute of Telecommunications (Inatel), 510 João de Camargo Av., Santa Rita Dusapukai 37540-000, MG, Brazil; leticiacarneiro@get.inatel.br (L.C.S.); egidio.neto@inatel.br (E.R.N.); elima@get.inatel.br (E.S.L.)

**Keywords:** energy efficiency, hazardous environments, industrial IoT (IIoT), Industry 4.0, optical fiber, power-over-fiber (PoF), wireless sensor network

## Abstract

We report the experimental implementation of optically-powered wireless sensor nodes based on the power-over-fiber (PoF) technology, aiming at Industrial Internet of Things (IIoT) applications. This technique employs optical fibers to transmit power and is proposed as a solution to address the hazardous industrial environment challenges, e.g., electromagnetic interference and extreme temperatures. The proposed approach enables two different IIoT scenarios, in which wireless transmitter (TX) and receiver (RX) nodes are powered by a PoF system, enabling local and remote temperature data monitoring, with the purpose of achieving an intelligent and reliable process management in industrial production lines. In addition, the system performance is investigated as a function of the delivered electrical power and power transmission efficiency (PTE), which is the primary performance metric of a PoF system. We report 1.4 W electrical power deliver with PTE = 24%. Furthermore, we carry out a voltage stability analysis, demonstrating that the PoF system is capable of delivering stable voltage to a wide range of applications. Finally, we present a comparison of temperature measurements between the proposed approach and a conventional industrial programmable logic controller (PLC). The obtained results demonstrate that PoF might be considered as a potential technology to power and enhance the energy efficiency of IIoT sensing systems.

## 1. Introduction

The fourth industrial revolution, so called Industry 4.0, is defined as the integration of complex physical automation systems composed by machinery and devices connected via sensors, which are controlled by software, aiming to improve process performance and reliability [1]. Key features of Industry 4.0 are digitization, automation, adaption, optimization and customized production. Thus, high-performance embedded systems, Internet of Things (IoT), cloud computing, big data, artificial intelligence (AI), and wireless networks have been considered fundamental technologies to enable a smart, flexible, safe, and efficient manufacturing process. In other words, information technology has been used to improve the management of manufacturing resources and quality of service (QoS) [2,3,4].

The concept of using IoT technologies in manufacturing, known as Industrial IoT (IIoT), is considered a potential technique to enable Industry 4.0 scenarios, providing interconnection of engines, power grids, and sensors to the cloud in the industrial environment. This connectivity is extremely important to allow that all data collected in the complex industrial physical environment is available real-time for the software applications in the network top layers. Therefore, the integration of IIoT and Industry 4.0 enables robust, faster, and most importantly, secure systems. However, the industrial environment brings some challenges for IIoT implementation, namely high dynamic environment, severe electromagnetic interference levels, high environmental temperature, and explosive and hazardous areas [5,6]. Furthermore, energy efficiency is a key aspect of IIoT systems, as massive sensors, devices, and machines consume a considerable amount of energy, increasing the carbon footprint [7,8]. Consequently, it is crucial to provide solutions to properly supply the required power to sensors and other devices, ensuring operation stability, safety and robustness in the industrial environment.

power-over-fiber (PoF) technology might be a potential solution to supply the required power to remote devices and sensors in industrial environments. This technique, firstly reported in 1978 [9], consists of transmitting power by means of an optical fiber. The key motivation for using PoF systems over conventional power distribution lines in hazardous environments is the demand for power supplies that are immune to electromagnetic interference, short circuits and sparks. In addition, PoF systems provide galvanic isolation, weight reduction, and resistance to corrosion, moisture and extreme temperatures, which are all inherent to optical fibers [10,11]. In this context, PoF may be considered as an attractive alternative to increase safety and reliability of industrial systems by means of replacing conventional power supplies, metallic cables, and batteries.

Recently, the PoF technology has greatly improved due to the advance of the photonics components. Power levels have increased from less than 1 W to over 40 W in the last 20 years [12]. In addition, simultaneous power and data transmission has become feasible with the development of novel optical fibers [13,14]. Consequently, PoF has been employed to power sensors or sensing systems in a wide range of applications, including high-voltage installations, IoT, and hazardous and industrial environments.

In particular, Wang et al. [15] reported a PoF-based wireless sensor system for hazardous environments, in which an optically-powered remote transceiver is responsible for receiving and transmitting the acquired data to a base station through an optical fiber. The PoF system was capable of delivering 151.4 mW. The combination of PoF and free space optics (FSO) techniques has been previously employed to power an electrical current sensor in high voltage transmission lines and over 240 mW of electrical power was obtained [16]. Regarding industrial applications, a cost-effective PoF approach has been developed to power a base station which can handle up to four sensor nodes and is connected to a PC via universal serial bus (USB). The delivered electrical power was 31 μW and the achieved overall efficiency was 2.5% [17]. López-Cardona et al. reported optically-powered smart nodes based on magnetic field monitoring, fire, and temperature/presence sensors aiming at IoT-based solutions for power grid stations. Over 340 mW was achieved with efficiency of around 10% [18]. Moreover, the Authors in [19] demonstrated a PoF-based system designed to power IoT sensors (temperature and humidity), attaining 279 mW of delivered electrical power with efficiency of 16.5%. Bassan et al. [20] employed PoF for voltage and current measurements in medium voltage distribution networks, reaching 80 mW of electrical power, which was used to power a low threshold laser, electronic circuits, and sensors.

**Figure 1:**
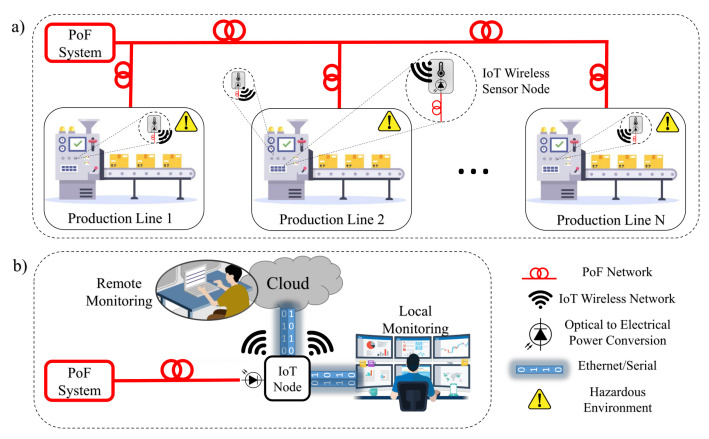
Optically-powered wireless sensor nodes for IIoT scenarios. (**a**) the first possible scenarios; (**b**) the second possible scenario.

Table 1 summarizes the main parameters of the state-of-the-art PoF solutions described in this section. One may note that most of the works are aimed at general IoT or hazardous scenarios, whereas the industrial scenario is poorly explored in the literature. In this context, aiming to achieve an intelligent and reliable process management in industrial production lines, we present the implementation of optically-powered wireless sensor nodes based on PoF technology for IIoT scenarios. Figure 1 illustrates the basic concept of this work. Local and remote data monitoring are enabled by the implementation of optically-powered IoT wireless transceiver nodes, which can be set to send or receive sensor data. Figure 1a depicts one of the possible scenarios, in which a PoF system is responsible for powering multiple IoT wireless sensor nodes composed of temperature sensors, control boards, and wireless transmitters (TXs). On the other hand, Figure 1b depicts the second possible scenario, in which the another PoF system drives the receiver (RX) node, responsible for receiving, processing, and sending the sensor data to the cloud or server by means of Ethernet or serial interface. Aiming to demonstrate the applicability of the PoF technology, our proof-of-concept is based on the experimental implementation of both scenarios, characterizing a realistic IIoT environment. As opposed to [15], which employed battery units to power wireless sensing nodes, we innovate by employing the PoF technology to replace conventional electrical wires and batteries in order to improve safety in hazardous environments. In addition, most works on PoF-based IIoT applications are focused on low power delivery [17], aiming to only drive low-complexity sensing nodes from industrial environments. To the best of our knowledge, we achieved the highest delivered electrical power considering the PoF state-of-the-art solutions for sensing applications, as demonstrated in Table 1.

To the best of the authors’ knowledge, this work presents an unprecedented system performance comparison between a PoF-based sensing system with a conventional industrial programmable logic controller (PLC), with the purpose of demonstrating the feasibility and potential of this approach for real IIoT scenarios. The evaluation methodology consisted in varying the temperature in a controlled environment and analyzing the data acquired from the optically-powered nodes and PLC. Furthermore, the PoF system is evaluated in terms of the delivered electrical power and overall efficiency. A voltage stability analysis is reported for the first time in the literature in order to demonstrate the flexibility and applicability of our PoF-based sensing system. The manuscript is organized as follows. Section 2 regards a comprehensive IIoT overview, emphasizing its definition and potential areas. Section 3 describes the PoF technology and its basic components, focusing on the main system limitations. Section 4 presents the implementation of our optically-powered wireless sensor node solution. Section 5 describes the PoF-based approach and reports an industrial PLC comparison methodology. Section 6 is concerning discussions on the experimental results. Finally, the conclusions and future works are outlined in Section 7.

## 2. IIoT—Industrial IoT

In contrast to IoT, which is aimed at consumer usage, IIoT is used for industrial purposes, e.g., manufacturing, supply chain monitoring, and management systems. Therefore, IIoT can be characterized as the connection of wireless sensor networks (WSNs) and wireless sensor and actuators networks (WSANs) in industrial environments aiming to collect data from different sensors, actuators and machines, focusing on transfer and control of critical mission information, which relies on machine-to-machine M2M communications [21,22]. In an IIoT environment, the data obtained from sensors, actuators and machines are analyzed to generate valuable information for factory operation and control devices. Extensive analysis of the industrial big data is typically performed by cloud, since the sensors, actuators, and machines generate different types of data in real time and update this data continuously [23].

Communication technologies are the base of IIoT, since manufacturing data sensed is transmitted and exchanged between different communication terminals to realize the interconnection of heterogeneous production factors. Communication technologies in network and application layers can be divided into wired, which include fieldbus and Ethernet technologies and wireless, which typically employ ZigBee, Wireless Fidelity (Wi-Fi), fourth-generation of mobile networks (4G), fifth generation of mobile networks (5G), and others [24].

In particular, industrial fieldbus technology is mainly used to provide communication between sensors, instruments, actuators, and other underlying field devices and perform the data transmission between the devices with upper level control system. Typical representatives are controler area network (CAN), ProfiBus and HART. On the other hand, industrial Ethernet aims to realize vertical integration between management systems and shop-floor, through the integration between the Ethernet transmission control protocol (TCP)/Internet protocol (IP) and industrial fieldbus. As a consequence, new protocols take place, as Modbus TCP, ProfiNet, and Ethernet/IP. A wide range of bus communication technologies provides reliable guarantee for communication between heterogeneous production factors in shop-floor and the upper control system. However, cross communication between different shop-floors mainly depends on the traditional Internet, which has the disadvantages of large delay, low security, and complex line layout [25].

WSNs have been widely explored in the literature and are considered as an effective approach to achieve ubiquitous connectivity in IIoT environments. Wireless communication allows users to easily connect mobile and inaccessible devices, simplifying line layout and reducing costs. Several wireless communication technologies have been employed in IIoT, including small-area wireless communication technologies as Wi-Fi, Zigbee, IPv6 over Low-Power Wireless Area Network (6LoWPAN), Bluetooth, and large-area wireless communication technologies as 4G and 5G. Small-area wireless communication technologies are not suitable for the wide range of IIoT. However, transmission rate, node connection, time-delay, reliability and security of the current large area wireless communication technologies are not enough to meet the demands of future IIoT [26].

The IIoT implementation also enables cyber physical manufacturing system (CPMS), which is designed to achieve smart management in manufacturing physical spaces, such as smart planning, production factor optimization, shop-floor optimization, production scheduling and inventory management [27]. Some functionalities are smart interconnection of shop-floor heterogeneous production factors [24], cloud computing and edge computing driven smart manufacturing [28], smart manufacturing based on virtual reality and augmented reality [29], digital twin driven smart manufacturing [30], and service-oriented to smart manufacturing technology [31].

The use of IIoT in hazardous environments is a key aspect to improve control, instrumentation and monitoring functionalities in automation and control systems. In this context, the use of IIoT technologies has increased considerably on the last few years, mainly in critical areas, where a secure operation system is mandatory [32]. Thus, the acquisition of data from different IIoT sensors in the industrial hazardous environment has enabled to differentiate the hazards, improving the system reliability [33]. From the energy-efficiency perspective, the increasing number of devices in an IIoT system lead to a considerable energy consumption. Therefore, energy efficiency is one of the most important challenges that needs to be addressed by using different techniques in different layers of the system, from the physical to the upper layers [34,35,36].

## 3. Power-over-Fiber (PoF)

**Figure 2:**
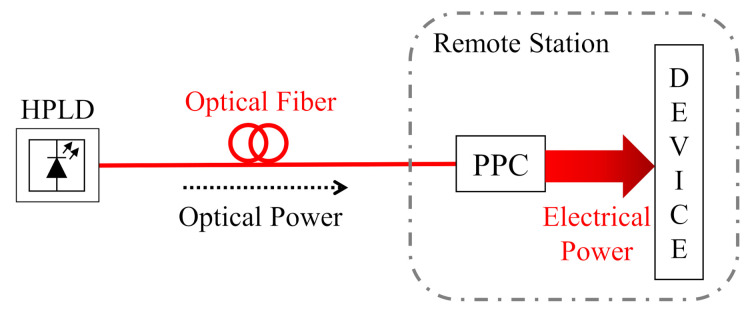
Block diagram of a generic PoF system. HPLD—high power laser diode; PPC—photovoltaic power converter.

The PoF technique basically consists of delivering electrical energy to a device at a remote location through an optical fiber [37,38]. Figure 2 depicts a block diagram of a generic PoF system. A high-power laser diode (HPLD) generates a high-power light, i.e., optical power signal, which is transmitted through an optical fiber link. The optical power is then converted into electrical power by a photovoltaic power converter (PPC), located at a remote station hundreds of meters away from the HPLD location [39,40].

The amount of delivered electrical power is determined by the HPLD output power, PPC efficiency, and optical fiber characteristics such as attenuation and length. The power budget of a generic PoF link is given by:

(1) $$P_{D}=\left(\frac{P_{\mathrm{HPLD}}\;\alpha_{\mathrm{link}}\;\eta_{\mathrm{PPC}}}{100}\right),$$

where $P_{D}$ is the delivered electrical power in W, $P_{\mathrm{HPLD}}$ is the HPLD output optical power in W, $\alpha_{\mathrm{link}}$ is the total optical fiber link attenuation, and $\eta_{\mathrm{PPC}}$ is the PPC conversion efficiency expressed as percentage.

One may note that the main challenges regarding the implementation of a PoF system are related to the HPLD output power, total optical fiber link attenuation, and the PPC conversion efficiency. The HPLD output power depends on the operating wavelength and electrical-to-optical (E/O) conversion efficiency. In addition, fiber type and length have great impact on the total link attenuation.

The HPLD is responsible for generating the optical power, which will subsequently be converted into electrical power. The HPLD operating wavelength and conversion efficiency are major concerns in PoF systems and mostly depend on the composition of the material. In particular, HPLDs commonly operate in the range of 808 to 980 nm [38,39]. The selected wavelength might impact on the total fiber link attenuation since shorter-wavelength results in a higher power transmission loss. Therefore, the optical link distance must be carefully evaluated in order to determine the most suitable wavelength range [40]. In addition, the HPLD output power is determined by the E/O conversion efficiency ($\eta_{\mathrm{HPLD}}$) which can be written as:

(2) $$\eta_{\mathrm{HPLD}}=\left(\frac{P_{\mathrm{HPLD}}}{P_{\mathrm{elec}}}\right)\;100,$$

where $\eta_{\mathrm{HPLD}}$ is expressed as percentage, $P_{\mathrm{HPLD}}$ is the HPLD output optical power, and $P_{\mathrm{elec}}$ is the input electrical power. The HPLD conversion efficiency typically ranges from 30% to 50% [40].

The main purpose of the optical fiber in a PoF system is to transmit as much power as possible from the HPLD to the PPC [39]. In this context, we emphasize two main aspects that can limit the amount of transmitted power, namely fiber attenuation and threshold power. Although fiber attenuation reduce the delivered power, it may not be a major concern for most of PoF systems that employ dozens of meters of fiber. For instance, a standard multimode fibers (MMF) attenuation is approximately 2.6 dB/km at 850 nm. However, it could be a problem for link lengths longer than 1 km, depending on the operating wavelength [38]. In addition, optical fibers present a threshold power ($P_{\mathrm{th}}$) which is defined as the highest amount of power that can be launched into the fiber without damaging it, which is given by [41]:

(3) $$P_{\mathrm{th}}=I_{\mathrm{th}}\;A_{\mathrm{eff}},$$

where the fiber power density threshold ($I_{\mathrm{th}}$) depends on the fiber composition and optical signal wavelength and lies between 1 and 5 MW/cm^2^ [42] and $A_{\mathrm{eff}}$ stands for the fiber effective mode area, which is defined as [41]:

(4) $$A_{\mathrm{eff}}=\pi \;{\left(\frac{d_{\mathrm{eff}}}{2}\right)}^{2},$$

where $d_{\mathrm{eff}}$ is the fiber effective diameter. The threshold power is directly proportional to the core effective area and, consequently, to the core effective diameter. Correspondingly, the larger the core effective diameter, the more power can be transmitted through the fiber. In this context, MMFs have been widely employed in PoF systems due to the large core diameter (typically from 50 to 200 μm) and compatibility with the HPLD wavelength bands [43,44,45,46]. On the other hand, single-mode fibers (SMFs), which are frequently employed in conventional high-data-rate transmission systems, may not be suitable to transmit high-power light due to its small core diameter (typically from 8 to 10 μm), which gives rise to critical damage to the fiber caused by the extremely high power density [40]. Other fiber types, such as double-clad fibers (DCFs) and multicore fibers (MCFs), have also been employed to enable simultaneous power and data transmission [41,47,48].

The PPC enables the conversion of the input optical power into electrical power and, therefore, is a critical component in a PoF system. In contrast to the conventional solar cells, PPCs must be compact to match the beam diameter generated from HPLD. Typically, PPC is made from a miniaturized circular solar cell array, usually 2 mm × 2 mm, having a circular aperture diameter of 1.5 mm. In addition, this component is typically designed to support very high-density optical power, contrarily to the conventional photovoltaic systems [11,37,49]. The most important performance indicator of a PPC is the optical-to-electrical (O/E) conversion efficiency, given by [38]:

(5) $$\eta_{\mathrm{PPC}}=\left(\frac{P_{\mathrm{elecmax}}}{P_{\mathrm{in}}}\right)\;100,$$

where $\eta_{\mathrm{PPC}}$ is expressed as percentage, $P_{\mathrm{elecmax}}$ is the maximum electrical power delivered by the PPC, and $P_{\mathrm{in}}$ is the incident high-power light, which must be compatible with the PPC maximum input power specifications. The PPC conversion efficiency varies as a function of the wavelength range and depends on the material composition, geometry, illumination intensity, and temperature. Most PPCs are developed for wavelength bands of 800 nm to 980 nm with efficiencies ranging from 30% to 60% [11,40].

The power transmission efficiency (PTE) is one of the key performance metrics in a PoF system. It is generally defined as the ratio between the HPLD output power and total electrical power delivered by the PPC or as defined by [48]:

(6) $$\mathrm{PTE}=\left(\frac{P_{\mathrm{HPLD}}}{P_{\mathrm{elecmax}}}\right)\;100,$$

where PTE is expressed as percentage, $P_{\mathrm{HPLD}}$ is the HPLD output optical power, and $P_{\mathrm{elecmax}}$ is the maximum electrical power delivered by the PPC. Equation (6) comprises all PoF components constraints, meaning that the PTE could be understood as the combination of the HPLD, optical fiber, and PPC overall efficiencies. Most works reported in the literature present PTE ranging from 10% to 40%.

## 4. Optically-Powered Wireless Sensor Nodes Implementation

This section describes the PoF implementation, envisaging its application to optically power wireless nodes aiming to enhance the control process and failure prevention in an IIoT environment. The PoF technique has been used to enable two distinct industrial scenarios, in order to demonstrate its flexibility to meet multiple applications. In the first scenario, the PoF system powered two IoT TX nodes, composed of temperature sensors, control boards and wireless TXs, establishing the connection with the RX node. The second scenario consisted in powering the IoT RX node, validating the PoF technique applicability for optically powering TX and RX IoT nodes. The system implementation analysis has been divided into three parts, namely PoF system description and analysis, first scenario, and second scenario implementation.

### 4.1. Power-over-Fiber (PoF) System Characterization

**Figure 3:**
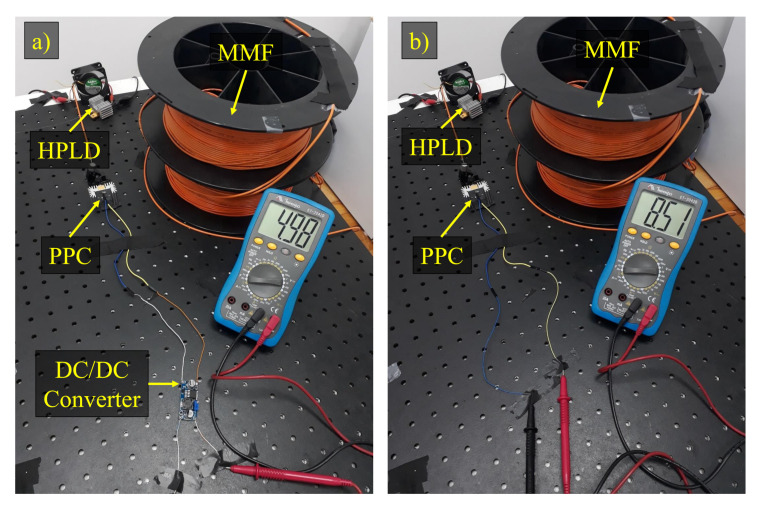
PoF system experimental implementation: (**a**) With the DC/DC converter. (**b**) Without the DC/DC converter. HPLD—high-power laser diode; MMF—multimode fiber; PPC—photovoltaic power converter.

Generally, PoF systems are composed of a HPLD followed by an optical fiber link and a PPC. In particular, our PoF experimental implementation, illustrated in Figure 3, consisted of a HPLD operating in continuous wave (CW) and centered at 975 nm with maximum emitted optical power of around 30 W. In the first scenario, 0.6 W optical power was required to power one TX node, whereas 1.5 W was sufficient to power two TX nodes. In the second scenario, the RX node demanded 6 W optical power from the HPLD. Subsequently, a 100 m MMF link was employed to transmit the optical power to the PPC. The link length was limited to 100 m, mainly due to the MMF high attenuation at shorter wavelengths, i.e., 4 dB/km at the HPLD center wavelength. On the other hand, the maximum PoF MMF link length reported in the literature was around 4 km [44]. Consequently, the designed PoF system may not be suitable to power a sensors at extreme long distances. Nevertheless, the 100 m MMF link is compatible with the dimensions of an industrial site and could be extended by employing higher transmitted optical power and fibers with lower attenuation. The fiber core and clad diameters are 100 μm and 140 μm, respectively. In addition, the PPC must also be compatible with high incident optical power. We have used a commercially available PPC (YCH-L300) from MH GoPower, which presents approximately 30% conversion efficiency and offers FC connectorization. However, the extreme high optical power density might cause damage to optical fiber connections, limiting the transmitted optical power [40]. Therefore, at the PoF reception, the light from the optical fiber was directly launched to the PPC input, so that the laser beam can illuminate all the PPC cell sectors, increasing the conversion efficiency. In the first scenario, a step-down DC/DC converter (LM2596) was necessary to regulate and reduce the voltage (see Figure 3a) as the TX node operates at 5 V and the PPC delivers 8.5 V output DC voltage (see Figure 3b). By contrast, the RX node operates at voltages ranging from 7 V to 12 V, thus the DC/DC converter was not required in the second scenario. Table 2 lists the detailed PoF system features.

**Figure 4:**
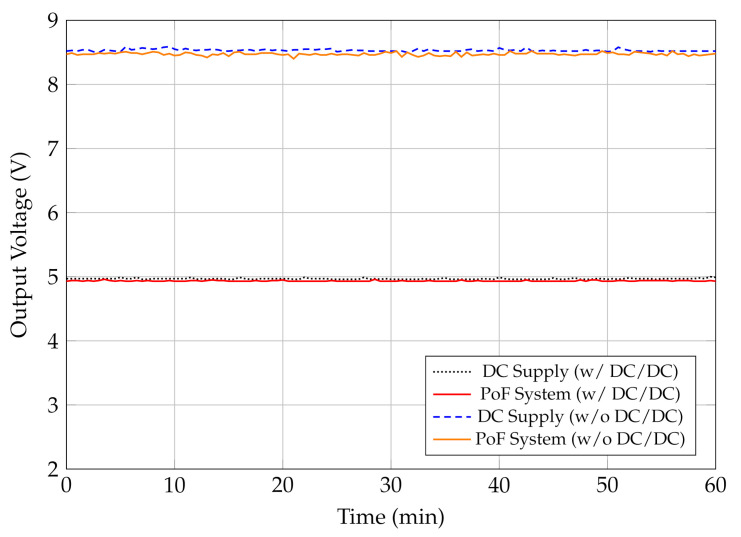
Stability comparison between PoF system and a conventional DC supply with and without the DC/DC converter at 5 V and 8.5 V, respectively, over 60 min.

Constant supply voltage and current are crucial parameters to ensure reliable and accurate performance of industrial sensing systems. In this context, we have compared the PoF system output voltage considering two scenarios: conventional DC supply (MPL-3305M MINIPA) and PoF system providing 5 V and 8.5 V, respectively. In the first case, we have employed a DC/DC converter in order to meet the requirements of the first scenario. Figure 4 depicts the output voltage measurements over a 60 min period. One may note that the voltage supplied by the PoF system is comparable to the DC supply measurements for both conditions: with or without the DC/DC converter. Although no significant voltage fluctuations are observed, the 8.5 measurements present slightly variations compared to the 5 V measurements. This contrast is due to the DC/DC converter which, in addition to reducing the output voltage, performs voltage regulation and stabilization. Nevertheless, the designed PoF system is capable of delivering stable output voltage with or without the DC/DC converter.

### 4.2. The First Characterization Scenario

**Figure 5:**
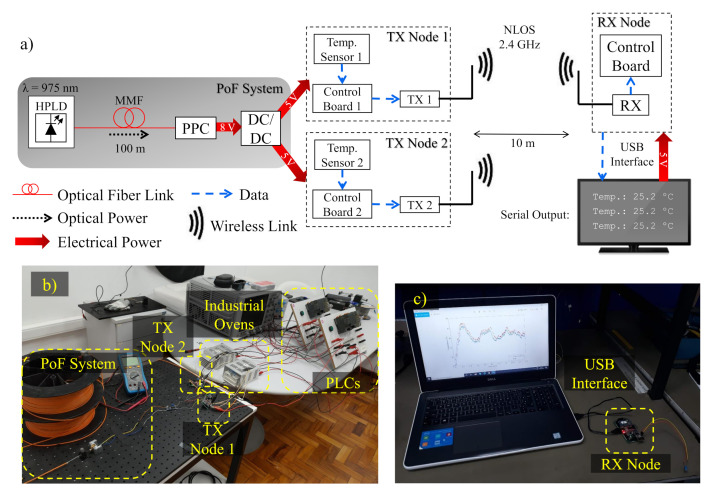
Optically-powered wireless TX sensor nodes implementation. (**a**) Experimental setup block diagram. (**b**) Experimental setup photograph of PoF system, TX nodes, industrial oven and PLC. (**c**) Experimental setup photograph of RX node, USB interface and the computer. HPLD—high-power laser diode; MMF—multimode fiber; PPC—photovoltaic power converter; TX—transmitter; RX—receiver; NLOS—non-line-of-sight; PLC—programmable logic controller.

Figure 5a depicts the block diagram of the first implemented scenario. The TX node is composed of a temperature sensor, a control board and a wireless TX. The employed low-cost temperature sensor (LM35) presents output voltage linearly proportional to the temperature in degrees Celsius. The sensor bias voltage and temperature range varies from 4 V to 30 V and from 0 ${}^{\circ }$C to 120 ${}^{\circ }$C, respectively. The control board consists of an Arduino Pro Mini microcontroller based on the microchip ATmega328P, which is responsible for controlling the main functions of the system, and a conversion module (FTDI FT232R), which performs the USB to transistor-transistor logic (TTL) serial conversion. The transceiver module (nRF24L01+) operates in the 2.4 GHz frequency band, which is an industrial, scientific and medical (ISM) band internationally reserved to unlicensed low-powered devices. The ISM applications include wireless computer networks (WiFi), Bluetooth devices and near field communication (NFC) [50]. The module employs Gaussian frequency-shift keying (GFSK) modulation with programmable data rate of 250 kbps, 1 Mbps, and 2 Mbps, and transmission output power of 0 dBm, −6 dBm, −12 dBm, and −18 dBm. Table 3 lists the detailed TX node features.

The first step was to power only the TX node 1, in order to evaluate the PoF system capabilities. In this scheme, over 0.6 W optical power was transmitted from the HPLD to the PPC. Considering fiber and splice attenuation, the total PoF link loss could be estimated in approximately 1 dB. Therefore, over 0.5 W optical power was injected into the PPC, which presents a conversion efficiency of approximately 30%, resulting in a delivered electrical power of around 152 mW. Finally, we measured the TX node 1 consumed power at the DC/DC converter output, which resulted in approximately 140 mW. Consequently, PTE of around 23% could be achieved with this configuration.

The control board 1 was responsible for processing and sending the acquired temperature data to the wireless TX 1, which transmitted the data every 100 ms. The wireless propagation was performed over a 10 m link at 2.4 GHz, attaining 2 Mbps and 0 dBm of output power, in a laboratory environment composed of computers, tables, glass doors, and pieces of equipment, characterizing a non-line-of-sight (NLOS) environment. The wireless link is relatively low-power, meaning the link distance between the TX and RX nodes is limited to a few meters. Nevertheless, longer-reach wireless links could be achieved by increasing the transmission power and RX sensitivity. In addition, the measurements have been obtained during business hours, with researchers and students at the lab, characterizing a realistic wireless scenario. At the reception side, the RX node, which consists of an identical control board and wireless transceiver, have been used to receive and process/decode the acquired temperature data. Finally, the temperature information was displayed by a computer via serial data transfer to provide real-time and local data monitoring. In this case, all the equipment at the reception side were powered by the computer USB port.

The second step was to add a second TX node in order to evaluate the system scalability. In this scheme, 1.5 W optical power was transmitted through the same fiber link, with attenuation of 1 dB. Thus, we estimated that over 1.2 W optical power was injected into the PPC, resulting in a delivered electrical power of around 360 mW. Finally, we measured the TX node 1 and 2 overall consumed power at the DC/DC converter output, which resulted in approximately 330 mW. Consequently, PTE of around 22% could be achieved with this configuration. The wireless TX 1 transmitted the acquired temperature data from sensor 1 every 100 ms, whereas the TX node 2 simultaneously transmitted the data from sensor 2 every 200 ms. The wireless transmission was performed in the same NLOS environment. At the reception side, the same RX node was employed and the data was displayed by the computer via serial data transfer. Figure 5b presents an experimental setup photograph, which includes the PoF system, TX nodes, industrial oven and PLC, whereas Figure 5c displays the RX node, USB interface and the computer.

### 4.3. The Second Characterization Scenario

**Figure 6:**
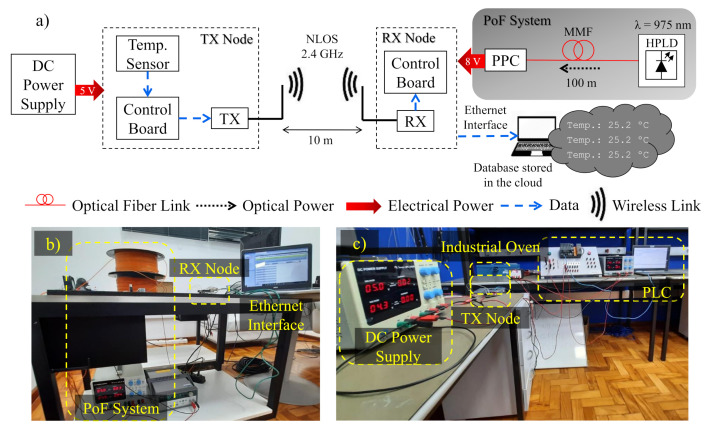
Optically-powered wireless RX node implementation. (**a**) Experimental setup block diagram. (**b**) Experimental setup photograph of the PoF system, RX node, Ethernet interface and the computer. (**c**) Experimental setup photograph of the DC power supply, TX node, industrial oven and PLC. HPLD—high-power laser diode; MMF—multimode fiber; PPC—photovoltaic power converter; TX—transmitter; RX—receiver; NLOS—non-line-of-sight; PLC—programmable logic controller.

The second characterization scenario consisted of employing the designed PoF system to power the IoT RX node, as described in Figure 6a. This scenario is based on the assumption that the TX node may require some mobility, which calls for a battery unit. However, safety and management systems must be taken into account in order to avoid explosion and accidents in an industrial environment. Therefore, we employed a DC power supply only for tests purposes. In this implementation, the TX node is composed of the same components employed in the first scenario: Arduino Pro Mini, serial converter, and wireless TX. On the other hand, the RX node consisted of the wireless transceiver module (nRF24L01+), an Arduino UNO which process the received data, and an Ethernet shield based on the chip W5100 (WIZnet), which allows the Arduino board to connect to the Internet by providing a network IP stack capable of both TCP and user datagram protocol (UDP). Table 4 lists the detailed RX node features.

In this scheme, 6 W optical power was transmitted through the same fiber link, with attenuation of 1 dB. Thus, we estimated that over 4.8 W optical power was injected into the PPC. In this case, the DC/DC converter was not required, since the 8.5 V voltage provided by the PoF system properly powers the RX node. Finally, we measured the RX node consumed power at the PPC output, which resulted in approximately 1.4 W due to the PPC 30 % efficiency. Consequently, PTE of around 24% could be achieved with this configuration. One may note that the RX node consumes more power than the optically-powered TX node employed in the first scenario. This difference is due to the Arduino UNO use, which is more complex than the Arduino Pro Mini, demanding more power. Moreover, the Ethernet shield typically consumes 100 mA, as shown in Table 4.

The wireless transmission was performed under the same characteristics of the previous scenario: frequency of 2.4 GHz; data rate of 2 Mbps; output power of 0 dBm; 10 m wireless link at a laboratory environment. At the receiver side, the data acquired from the RX module is sent to Arduino UNO, which processes and sends the data to a web server via Ethernet interface. Basically, we have implemented a data logger, which allows for continuous real-time data logging and monitoring, providing the users comprehensive information about the condition of the equipment being monitored. In addition, data can also be recorded and stored in a database for online or even offline local or remote monitoring. The implemented database was built on an open-source software tool written on PHP (phpMyAdmin), which enables the administration of MySQL database servers over the Web [51]. Figure 6b presents an experimental setup photograph, which includes the PoF system, RX node, Ethernet interface, and the database displayed in the computer, whereas Figure 6c displays the DC power supply, TX node, industrial oven and PLC.

## 5. Power-over-Fiber (PoF) and Industrial PLC Comparison Methodology

In this Section, we present the methodology used to evaluate the temperature sensor performance, by means of a comparison between the designed PoF-based approach and conventional industrial sensing system, with the purpose of evaluating our approach feasibility and potential. In this context, a PLC have been employed, which is basically a computer-based device designed to control many types of industrial pieces of equipment and automated systems. Generally, PLCs are composed of five main blocks, including rack assembly, power supply, programming device, input/output section, and central processing unit (CPU) [52]. The comparison consisted in varying the temperature in a controlled environment and analyzing the data acquired from the optically-powered nodes and PLC. In particular, an industrial oven was employed to emulate the temperature controlled environment. The analysis consisted in compiling temperature measurements over a defined time period and comparing the resulting PoF-based approach and PLC curves. Accordingly, we have explored the two aforementioned scenarios: temperature data acquired by one or two optically-powered TX nodes, sent to the RX node over a wireless link, and stored in a computer via serial interface; temperature data acquired by a TX node powered by a DC supply, sent to the optically-powered RX node over a wireless link, and stored in a database via Ethernet interface.

**Figure 7:**
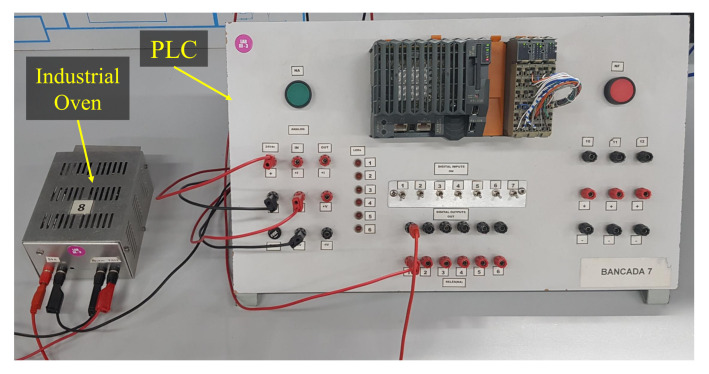
Photograph of the PLC and industrial oven employed for comparison purposes.

We have employed a didactic plant that emulates an industrial oven, where the temperature sensor was placed. The oven temperature is controlled by a proportional-integral-derivative (PID) controller embedded in the PLC, as presented in Figure 7. The oven is composed of a resistor, which converts electrical energy into thermal energy, and a solid state relay (SSR) for ON/OFF control. The temperature inside the oven is converted into voltage by the sensor in order to be read by the PLC analog input. The PID controller algorithm embedded in the PLC performs the closed-loop control based on the temperature set-point, defined by the user, and the real time temperature is known as the process value. The PID controller output variable is proportional to the duty cycle of a pulse width modulation (PWM) signal, which controls the SSR switch, responsible for controlling the resistor dissipated average power that works as an actuator in the heat system.

In this scheme, the temperature sensor (LM35) is connected to the oven as an additional sensor which is not used to close the control loop. Aiming to efficiently compare the data acquired directly from the PLC analog input to the data acquired from the optically-powered sensor node, we have employed a temperature sensor with the same characteristics of the temperature sensor employed in the closed-loop. However, no digital filters were enabled in this case. In addition, the control system response is a typical under-damped second order response, which has been used in all temperature measurements. Therefore, the proposed PoF-based IIoT system works in addition to the conventional industrial system, collecting field data which can be used in big data and analytics solutions to improve maintenance, safety and productivity.

## 6. Experimental Results and Discussion

**Figure 8:**
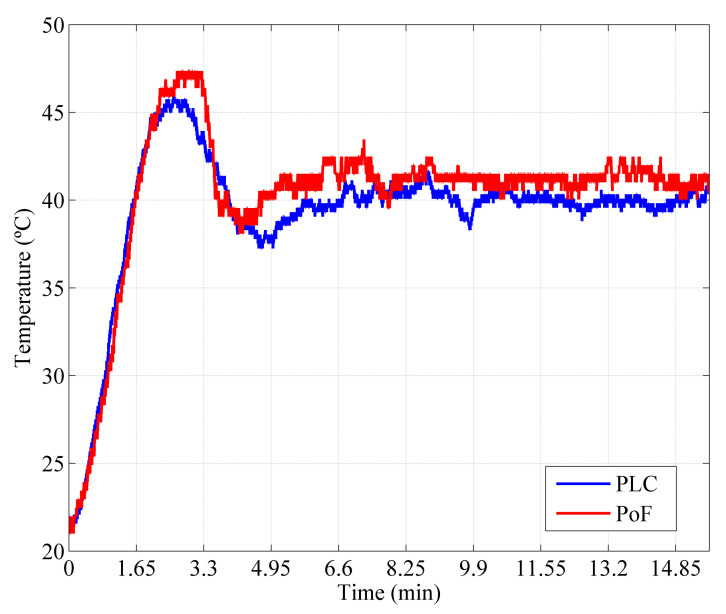
Temperature comparison between the PoF-based approach and the conventional industrial PLC. The temperature measurements were acquired from the optically-powered TX node 1 (first scenario).

This section presents the experimental results and discussion regarding the temperature sensor performance comparison between the designed PoF-based approach and industrial PLC. The first analysis consisted in comparing the acquired temperature data from the optically-powered TX node 1 and conventional industrial PLC, representing the first implemented scenario. Figure 8 presents the temperature measurements over a 17.2 min period. The initial temperature inside the oven was 25 ${}^{\circ }$C and a set-point of 40 ${}^{\circ }$C was defined to perform the experimental measurements. In both cases it is possible to observe a settling time of 15.5 min and a temperature oscillation mainly caused by the external disturbance in the environment. However, a temperature peak of 47 ${}^{\circ }$C was observed in the optically-powered TX node 1 measurements, whereas the PLC temperature peak was 45.5 ${}^{\circ }$C. This divergence may occur due to three main differences between the Arduino used in the TX node 1 and the industrial PLC: analog-to-digital converters (ADCs) resolution; electromagnetic compatibility and robustness; voltage at the analog inputs. In particular, Arduino analog voltage input operates from 0 to 5 V, which is converted to a decimal value ranging from 0 to 1023, as it uses 10 bits for encoding. The resolution in this case can be determined by:

(7) $$R_{\mathrm{ARD}}=\left(\frac{5-0}{1023-0}\right),$$

resulting in 4.888 mV. The PLC analog voltage input operates from 0 to 10 V, which is converted to a decimal value ranging from 0 to 32,767, as it uses 15 bits for encoding and 1 bit for parity, totaling 16 bits. The resolution in this case can be determined by:

(8) $$R_{\mathrm{PLC}}=\left(\frac{10-0}{32767-0}\right),$$

resulting in 305.185 μV. As a consequence, slightly different temperature values are acquired by the Arduino and PLC, impacting on the obtained curves. It is worth mentioning that the Arduino and PLC are different in terms of computational architecture, including memory capacity, processors and other elements. In addition, the PLC is compatible and designed specifically for industrial applications, consequently less susceptible to electromagnetic interference, compared to Arduino.

Although the same temperature sensor was connected to the PLC and Arduino analog inputs, the devices were powered by different DC supplies. The PLC is powered by an independent 24 VDC supply, whereas Arduino and the temperature sensor were powered by the PoF system. In this case, the common ground pin of all devices were connected in order to balance the voltages and provide the same reference frame. Nevertheless, there may be a difference in received voltage at the analog input of the devices at a certain time and at a certain temperature in the oven. Another source of interference that can generate a potential difference between the voltages received by the analog inputs of the devices is caused by the Seebeck effect, which occurs when two junctions of different conductors or semiconductors are subjected to different temperatures [53].

**Figure 9:**
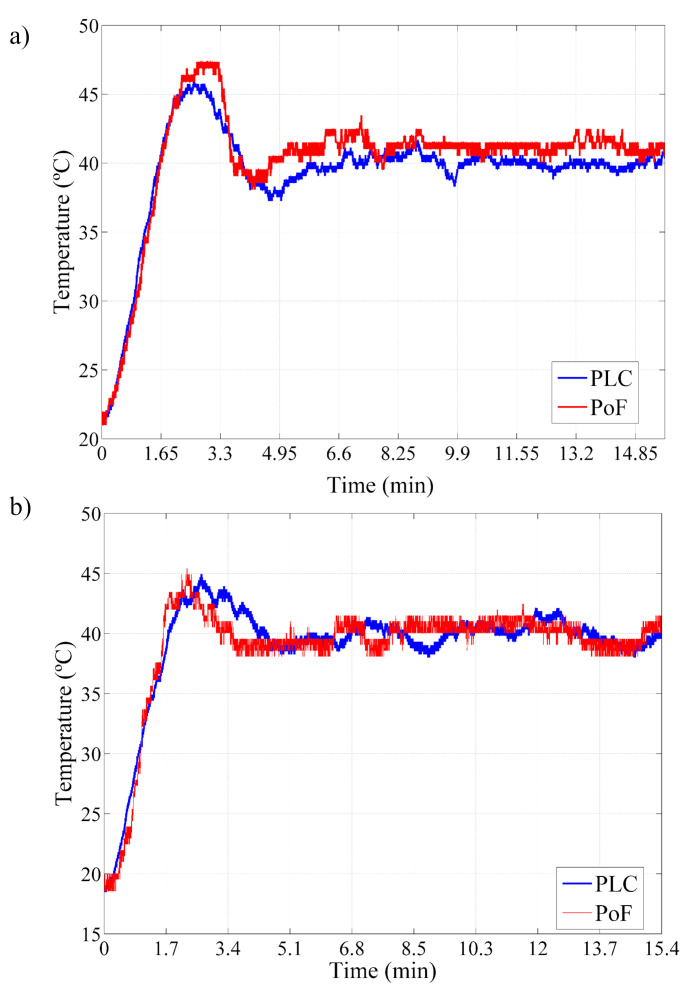
Temperature comparison between the PoF-based approach and the conventional industrial PLC (first scenario): (**a**) Temperature measurements acquired from the TX node 1. (**b**) Temperature measurements acquired from the TX node 2.

The next comparison consisted in performing the temperature measurements of two optically-powered TX sensor nodes transmitting simultaneously. In this case, we have employed two separate industrial ovens, where two different temperature sensors were placed (sensors 1 and 2). In addition, two independent PLCs were employed in order to enable the simultaneous data acquisition. Figure 9a presents the comparison between the acquired temperature data from the optically-powered TX node 1, which employs sensor 1, and the conventional industrial PLC. The initial temperature into the oven and set-point have been defined as 21 and 40 ${}^{\circ }$C, respectively, and a settling time of around 9.9 min could be observed for both systems. In addition, a temperature peak of 47.5 ${}^{\circ }$C was observed in the optically-powered TX node 1 measurements. By contrast, the PLC reported a temperature peak of 45.3 ${}^{\circ }$C. Regardless of this divergence, the optically-powered TX node 1 presents a very similar performance to the industrial PLC.

The TX node 2 and industrial PLC temperature measurements as a function of time are presented in Figure 9b. In this case, the initial temperature inside the oven and the set-point have been properly set to 19.5 ${}^{\circ }$C and 40 ${}^{\circ }$C, respectively. One may note extremely similar temperature peaks of approximately 45 ${}^{\circ }$C in both curves. In addition, both systems present a settling time of 6.8 min and slight temperature fluctuations which occur due to the external disturbance on the environment. Once again, the PoF-based approach and PLC present good agreement, demonstrating the PoF applicability and reliability to power IIoT sensor nodes with the remarkable advantage of optically-power the remote nodes.

**Figure 10:**
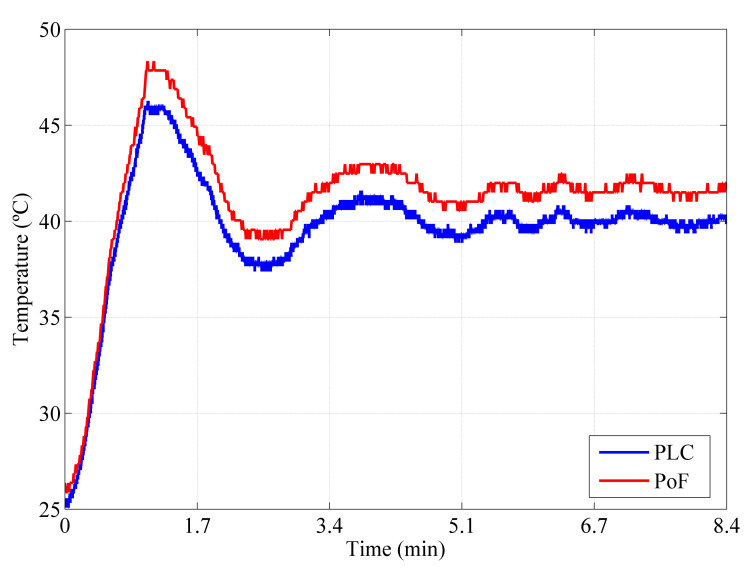
Temperature comparison between the PoF-based approach and the conventional industrial PLC. The temperature measurements were acquired from the TX node and sent to the optically-powered RX node (second scenario).

Regarding the second implemented scenario, the last comparison consisted in performing the temperature comparison considering the acquired temperature data when the TX node was powered by the DC supply and the designed PoF system was responsible for powering only the RX node. Figure 10 compares the temperature waveforms from the PoF-based approach and industrial PLC. The initial temperature inside the oven was 25 ${}^{\circ }$C and a set-point of 40 ${}^{\circ }$C was configured to perform the measurements. The measurements performed by the optically-powered system presented a temperature peak of 48 ${}^{\circ }$C, whereas the PLC reported a temperature peak of 45.5 ${}^{\circ }$C. Both systems presented a settling time of 6.7 min. Although the curves follow the same fluctuation pattern, a temperature measurement divergence of approximately 2.5 ${}^{\circ }$C could be observed, which may be caused by the differences between Arduino and the PLC, including ADC resolution, electromagnetic compatibility and robustness, and voltage at the analog inputs. In addition, the wireless transmission performed in the PoF-based system may also contribute to this divergence, due to the NLOS environment characteristics. Nevertheless, the PoF- and PLC-based IIoT systems presented similar temperature measurements over the time, emphasizing the PoF feasibility to fully power IIoT sensor networks in hazardous environments.

## 7. Conclusions

We have successfully proposed, implemented and characterized a PoF-based sensing system composed of optically-powered wireless sensor nodes aiming at IIoT scenarios. The proposed PoF system consisted of a 975-nm HPLD, a 100 m MMF link, a 30% efficiency PPC, and a step-down DC/DC converter. In order to evaluate the PoF system applicability for powering IoT wireless nodes, we have carried out a voltage stability analysis and compared the obtained results with a conventional DC supply. Our PoF system was able to deliver stable output voltage, with or without the DC/DC converter, at 5 V and 8.5 V, respectively.

Furthermore, we have implemented two possible IIoT scenarios to demonstrate the flexibility and potential of the PoF technology for powering sensing systems. The first implemented scenarios consisted in powering multiple IoT wireless sensor nodes, composed of temperature sensors, control boards and wireless TXs. The temperature data was sent over a 10 m wireless link to an RX node, responsible for receiving, processing, and displaying the data by means of a serial interface. In this scheme, over 0.6 W optical power was transmitted and approximately 152 mW electrical power was delivered to power one TX node, resulting in a PTE of around 23%. On the other hand, when powering two TX nodes, 1.5 W optical power was transmitted, 330 mW electrical power was delivered, and PTE of around 22% was achieved.

The second implemented scenario consisted in optically-powering the RX node. In this experiment, the TX node was powered by a DC supply for tests purposes and the DC/DC converter was not required. The optically-powered RX node was capable of processing and sending the temperature data to a database stored in the cloud via Ethernet interface. In this scheme, 6 W optical power was transmitted and over 1.4 W electrical power was delivered, resulting in PTE of 24%. The results demonstrated that the PoF system is capable of providing the required power for less and more complex IoT wireless nodes, enabling an intricate WSN.

In order to evaluate the designed PoF-based sensing system performance, we have carried out an investigation based on a comparison between the proposed approach and a conventional industrial PLC. The comparison consisted in varying the temperature in an industrial oven and analyzing the data acquired from the optically-powered nodes and the PLC. We have considered the two implemented scenarios, in which the TX nodes and RX node were powered by the PoF system, respectively. The PoF- and PLC-based approaches presented similar temperature measurements over the time for both scenarios. In other words, the proposed PoF-based approach presented excellent performance compared to the industrial PLC, demonstrating that optically-powered IoT wireless nodes could be implemented in a realistic industrial environment.

Future works regard the implementation of more IoT wireless sensor nodes, envisaging a more complex and dense WSN installed in a real industrial environment, as well as improving and enhancing the PoF system efficiency and transmission distance. Further experimental evaluations concern the implementation of a different architecture based on media converters aiming to transmit data between optically-powered IoT nodes over an optical fiber link, as well as including a performance investigation and comparison with the wireless link.

## Figures and Tables

**Table 1:** Main parameters of the PoF state-of-the-art solutions for sensing applications.

Ref.	Application	Transmitted Optical Power (W)	Delivered Electrical Power (mW)	Transmission Distance (m)	Overall Efficiency (%)
[15]	Hazardous Environments	2	151.4	2000	No Data
[16]	High-voltage Installations	No Data	240	No Data	No Data
[17]	Industrial Environments	0.00125	0.031	20	2.5
[18]	IoT for Power Grids	1.5	340	300	10
[19]	IoT for Hazardous Environments	1.7	279	300	16.5
[20]	High-voltage Installations	2	80	No Data	No Data
This Work	Industrial IoT	6	1400	100	24

**Table 2:** PoF System Features.

Component	Specifications
Fiber-coupled HPLD	Center wavelength: 975 nm Maximum output power: 30 W
Optical Fiber	Type: MMF/ Length: 100 m Attenuation: 4 dB/km 100 μm/140 μm core/cladding diameters
PPC (YCH-L300 with Passive Heatsinking)	Operating wavelength: 915–980 nm Conversion Efficiency: 30%
DC/DC converter step-down (LM2596)	Input/output voltage: 3.2–40 V/1.5–35 V Conversion Efficiency: 92%

**Table 3:** TX Node Features.

Component	Specifications
Arduino Pro mini	Operating voltage: 5 V Max. Current Consumption: 5 mA
Conversion module (FT232R)	Operating voltage: 5 V Current Consumption: 15 mA
Transceiver module TX (nRF24L01+)	GFSK modulation Operating voltage: 1.9–3.6 V Operating frequency: 2400–2525 MHz Maximum transmission power: 0 dBm Current consumption: 11.3 mA at 0 dBm
Temperature sensor (LM35)	Operating voltage: 4–30 V Current Consumption: less than 60 μA Accuracy: ±1 ${}^{\circ }$C

**Table 4:** RX node features.

Component	Specifications
Arduino UNO	Operating voltage: 7–12 V Max. Current Consumption: 98 mA
Ethernet shield (W5100)	Operating voltage: 3–5.5 V Current Consumption: 100 mA
Transceiver module RX (nRF24L01+)	GFSK modulation Operating voltage: 1.9–3.6 V Operating frequency: 2400–2525 MHz Current consumption: 13.5 mA at 2 Mbps Sensitivity: −82 dBm at 2 Mbps
